# Stand-Alone Wearable System for Ubiquitous Real-Time Monitoring of Muscle Activation Potentials

**DOI:** 10.3390/s18061748

**Published:** 2018-05-29

**Authors:** Ivan Mazzetta, Paolo Gentile, Marco Pessione, Antonio Suppa, Alessandro Zampogna, Edoardo Bianchini, Fernanda Irrera

**Affiliations:** 1 Department of Information Engineering, Electronics and Telecommunications DIET, “Sapienza” University of Rome, via Eudossiana 18, 00184 Rome, Italy; ivan.mazzetta@gmail.com (I.M.); p.gentile7@gmail.com (P.G.); 2 ABB Italy Electrification Products, Via Pescaria 5, 24123 Bergamo, Italy; paolo.gentile@it.abb.com; 3 STMicroelectronics, Via Olivetti 2, 20864 Agrate Brianza MI, Italy; marco.pessione@st.com; 4 Department of Human Neuroscience, “Sapienza” University of Rome, Piazzale Aldo Moro, 00185 Rome, Italy; antonio.suppa@uniroma1.it (A.S.); alessandro.zampogna@gmail.com (A.Z.); edoardo.bianchini.92@gmail.com (E.B.); 5 IRCSS-NEUROMED, via Atinense 18, 86077 Pozzilli, IS, Italy

**Keywords:** wearable device, stand-alone processing, surface electromyography, embedded system, long-time monitoring

## Abstract

Wearable technology is attracting most attention in healthcare for the acquisition of physiological signals. We propose a stand-alone wearable surface ElectroMyoGraphy (sEMG) system for monitoring the muscle activity in real time. With respect to other wearable sEMG devices, the proposed system includes circuits for detecting the muscle activation potentials and it embeds the complete real-time data processing, without using any external device. The system is optimized with respect to power consumption, with a measured battery life that allows for monitoring the activity during the day. Thanks to its compactness and energy autonomy, it can be used outdoor and it provides a pathway to valuable diagnostic data sets for patients during their own day-life. Our system has performances that are comparable to state-of-art wired equipment in the detection of muscle contractions with the advantage of being wearable, compact, and ubiquitous.

## 1. Introduction

Application of Information Communication Technology (ICT) and sensor technology to healthcare is an innovative strategy for possibly improving the overall clinical management of specific diseases, especially in situations in which the conventional methods of medicine are poorly effective.

In this scenario, wearable devices and body sensor networks are attracting the most attention for those diseases requiring continuous monitoring for optimal therapeutic strategies [1,2,3,4,5,6,7,8,9,10]. The ultimate goal of wearable sensors is the convergence in a compact platform of multimodal recognition of distinct biological signals, such as electroencephalography, electrooculography, electromyography, electrocardiography for continuous, and automatic monitoring of human health status, thus possibly leading to improvement in the diagnosis, follow-up, and therapeutic strategies of several disorders. Moreover, the combined use of wearable sensors and Internet of Things (IoT) is opening to a variety of new applications with distributed virtual services based on embedded and cyber-physical systems [11].

Among others, the electromyography (EMG) is a well-known standardized technique that detects the muscle activity using needles or patch electrodes. It is extremely useful in those cases in which it is necessary to monitor the muscle activity and to detect/distinguish involuntary from voluntary muscle contractions, as in the case of specific movement disorders that are associated to neurological disease [12]. The surface EMG (sEMG) is not invasive, as opposed to the EMG with needles, and it is more comfortable and tolerated by patients. Conventionally, it is performed during outpatient visits by means of wired research laboratory equipment. It uses three patch electrodes that are properly placed on the skin. Positive and negative electrodes are positioned over the target muscle, whereas the third reference electrode has to be positioned far from it. Multichannel operation is possible, in order to acquire signals from more than one muscle at the same time. Examples of multichannel systems are reported in [13,14], where electrodes are positioned all around the forearm for hand motion recognition. In [14], an innovative two electrode configuration is proposed, sharing one electrode in adjacent channels.

**Figure 1:**
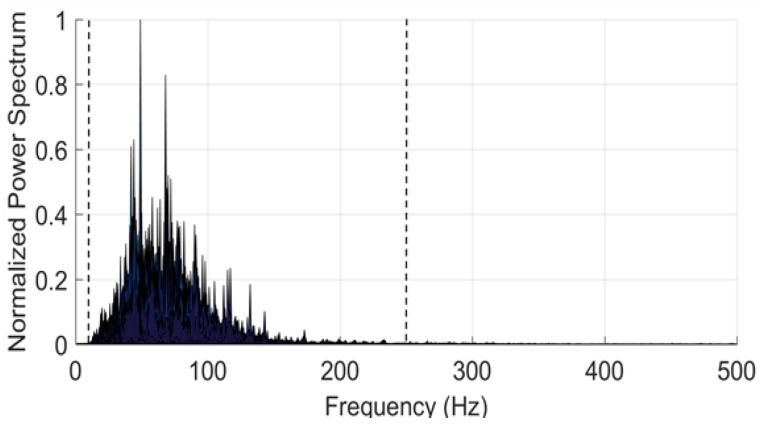
Sketch of the power spectrum of a typical raw electromyography (EMG) signal.

The power spectrum of a typical raw EMG signal is sketched in Figure 1. As one can see, the frequency components are typically in the band between 3 and 500 Hz, with a main content between 10 and 250 Hz (outlined with dashed vertical lines). This holds, regardless of the muscle and of the type of physical connections (needles or patch electrodes). In the case of sEMG, the signal amplitude is typically a few mVs or below (it rises up to 5.0 mV in special cases as for athletes).

The fact that, traditionally, the sEMG is performed in a health facility by means of wired equipment poses a number of problems. First, the test is short-lasting and is confined in a limited space, thus precluding lasting and free-living monitoring of specific symptoms in movement disorders [15]. Second, the presence of wires implies the execution of a limited range of movements, not allowing for the analysis of coordinated movements requiring wide change of body position, which is a major clinical issue [16]. Third, the wired connection to the electrical grid introduces noise at 50 Hz, in the mid of the most meaningful portion of the EMG spectrum [17,18].

In order to overcome the limitations of the wired conventional equipment, recently, wearable sEMG devices have been proposed and a few products are already available on the market. Most of the commercial products need an external signal processing unit [19,20,21,22], others take advantage of the integrated inertial units for classifying specific movements (as, for example, [23], which is used for hand gestures). Several research applications of those products can be found in the literature. For instance, in [24], the contraction frequency of the levator palpebrae muscle is monitored to control car drivers drowsiness; in [25], the hand gestures reproducing the sign language is detected; in [26], the sit-to-stand condition is monitored using a sEMG device on the quadriceps and a smartphone on the back to detect the trunk tilt angle. In [27], the authors realized a device in order to study the involuntary activity of the jaw muscles along the day. In [28], a wearable device is designed, which uses two electrode pairs for reading positive expressions from facial EMG signals. In [29], a network of several nodes that are distributed on the human body detects contemporarily electromyograms, electrocardiograms, accelerations, temperature, and input impedance. In all of those applications, data are processed on an external device (PC, smartphone, Arduino).

Unlike the mentioned wearable systems, the one that is proposed here performs the sEMG signal processing on-board and it recognizes in real-time the muscle activity without any external elaborating station. This is a considerable improvement since it opens to outdoor long term monitoring, allowing for the reliable evaluation of muscle activity during daily life, especially in patients with episodic and unpredictable disturbances, such as motor fluctuations and Freezing of Gait in Parkinson’s disease [30,31,32], or with motor impairment after stroke [33]. The lasting and objective evaluation of motor disturbances by means of wearable sEMG would significantly improve therapeutic management in the clinical setting, by overcoming the biased recall of symptoms self-report by patients [34]. The proposed system starts from a prototypal device that was developed by STMicroelectronics (Agrate Brianza, Italy), which is able to detect sEMG signals [35]. With respect to that preliminary work, in this paper, we extensively discuss the potentiality of that wearable system and present further systematic results that are related to a variety of muscle activities, including the least and short contraction and stretching. Our system is designed for low power-consumption with data saving on an integrated nonvolatile solid-state Flash memory making possible to monitor the muscle activity of the patient during his day-life. All of these improvements candidate our system as a valuable tool for collecting diagnostic datasets in domestic environment and outdoor.

## 2. Materials and Methods

The system recognizes the muscle activity without any external working station, since the processing algorithms run on the computational unit integrated on-board. In this operation mode, all of the information is recorded on the microSD. In alternative, data can be transmitted via Bluetooth to a PC. The system can eventually give a feedback in real time switching on an alarm (audio, LED, other) in specific cases. This option can be particularly useful in outpatient visits, when the doctor needs to check the activity intensity or time duration with respect to the determined thresholds and/or the eventual contraction of a specific muscle during movement disorders.

**Figure 2:**
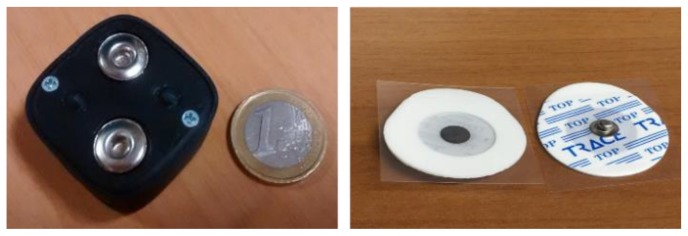
Back-side of Bio2Bit Move and the electrodes.

**Hardware**. The hard device is the prototypal device Bio2Bit Move (STMicroelectronics, Agrate Brianza, Italy) that was developed by STMicroelectronics, as shown in Figure 2. It includes: an ultra-low power bio-potential acquisition system with one differential channel for EMG acquisition (ST HM121), a 32 bit computational unit ARM^®^ Cortex^®^-M4, a microSD, a low-energy Bluetooth (BT) 4.0, a 592 mWh battery, a micro-USB connector. The Bio2Bit Move also includes a set of other physical and physiological sensors (not used in this work): electrocardiogram (ECG), Galvanic Skin Response (GSR), bioimpedance, accelerometer, gyroscope, and magnetometer. The presence of several functionalities in the same device opens to a possible future convergence of multimode operations on the same platform, with the contemporary detection of different biological and physical signals. The device dimensions are 30 × 30 × 15 mm, and the weight is 10 g, including the battery. Two clips are integrated into the device package (as shown in Figure 2). The electrode patches that are pictured in Figure 2 are buttoned in the clips so that their position remains fixed. The distance between the electrodes is 20 mm respecting the specification of SENIAM (a European concerted action in the EU Biomedical Health and Research program). Actually, the presence of fixed clips on the back of the device is an advantage with respect to measurements that were performed with conventional wired equipment. In fact, in this way, the distance between the two electrodes is always constant and the positioning of the adhesive electrodes can be performed by non-specialized users in a domestic environment, after they are trained to recognize specific muscles landmarks. Once the electrodes are fixed on the skin, in principle, the device adheres without falling down, thanks to its minimum weight (10 g). In any case, in practical long-time applications, it can be further tightened by elastic or cloth bands.

**Figure 3:**
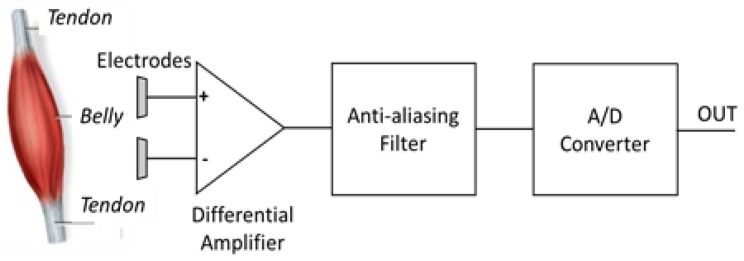
Block diagram of the bio-potential system (HM 121) used for stand-alone wearable surface electromyography (sEMG) signal acquisition.

The signal that is detected by patch electrodes needs to be amplified because of the attenuation due to the skin and the subcutaneous tissue. The block diagram of the bio-potential system (HM 121, STMicroelectronics, Agrate Brianza, Italy) for sEMG signal acquisition is displayed in Figure 3. The maximum amplification provided by HM 121 is 128, whereas the Common Mode Rejection Ratio (CMRR) (at 50 Hz) and the Signal to Noise Ratio (SNR) (with a signal of 10 Hz, 10 mVpp, gain 64) are respectively 65 dB and 59 dB. The integrated bandpass filter features bandwidth 0.5–300 Hz, to avoid aliasing effects and to eliminate the DC component. The integrated ADC features 14 bit resolution and ±0.6 V reference voltage. The measured overall noise is 38 μV, which is in agreement with requirements for medical applications (<50 µV). The device collects data in real time with a sampling frequency of up to 4 KHz.

**Software**. The choice of algorithm category depends on whether it is required that the processed signal is known a priori or not. To be more general, we decided to implement an algorithm independent of the a priori knowledge, because it gives greater flexibility and it is not related to any specific muscle. In fact, in the case of voluntary muscle contractions, the EMG signal is stochastic and it denotes a sudden variation of both amplitude and frequency, deriving from the activation of the action potential. Therefore, we focused onto the Teager-Kaiser Energy Operator (TKEO), which puts in evidence the instantaneous increase of the action potential and reduces the baseline noise [36]. In its discrete form in time domain, the TKEO is defined as:

(1) $$\Psi \left[x\left(n\right)\right]=x^{2}\left(n\right)-x\left(n+1\right)x\left(n-1\right)$$

where n is the sequence index. Applying the TKEO to a given signal with amplitude A, frequency $\omega_{0}$, and initial phase ϑ, of the type:

(2) $$x\left(n\right)=A\left(n\right)\cos\left(\omega_{0}\left(n\right)+\vartheta \right)$$

it becomes:

(3) $$\Psi \left[x\left(n\right)\right]=\;A^{2}\left(n\right)\sin^{2}\left(\omega_{0}\left(n\right)\right)$$

**Figure 4:**
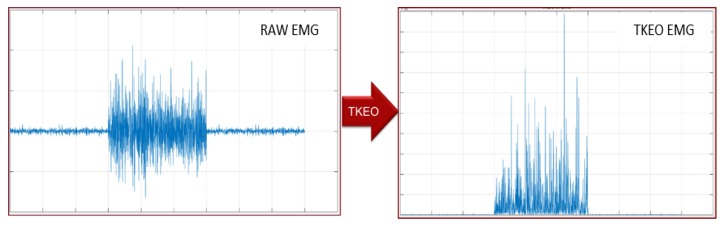
Raw and Teager-Kaiser Energy Operator (TKEO) EMG signal, on the same time and amplitude scale.

Therefore, the output of TKEO is positive and proportional to the product between the instantaneous amplitude and the frequency of the input signal. An example of TKEO operation is shown in Figure 4.

**Figure 5:**
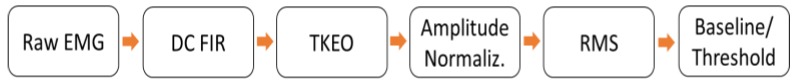
Data processing chain.

The threshold algorithm for detecting muscle contraction was then implemented in the TKEO domain, taking into consideration the standard minimum period of muscular contraction (50–70 ms), the standard minimum period of muscle inactivity (50–70 ms) and the margin of accuracy in the estimation of such measurements (±10 ms) [37,38]. The signal processing chain is shown in Figure 5.

In order to remove movement artifacts, the raw sEMG signal is high-pass filtered by a HP Finite Impulse Response (FIR) with a cut off frequency of 3 Hz. This filter subtracts the average of 32 previous samples to the current one. The subsequent steps consist on the TKEO rectification, the amplitude normalization, and the Root Mean Square (RMS) smoothing. The last step is the identification of the signal baseline and the definition of a dynamic threshold (T), which distinguishes the time intervals during which the muscle is active (ON state) from those during which it is inactive (OFF state). It holds:

(4) T=mean(EMG(tw))+j·std(EMG(tw))

**Figure 6:**
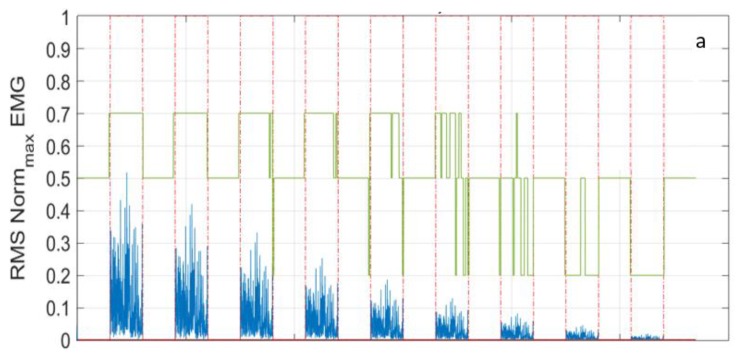

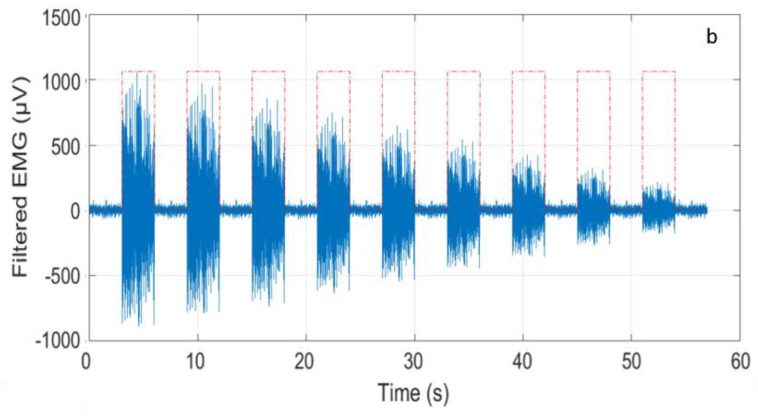
(**a**) The Root Mean Square (RMS) signal (blue) and factor *j* (green); and, (**b**) an example of the filtered EMG raw signal.

In the equation above, *mean* is the average value, *std* is the standard deviation, *j* is a dynamic factor, and *tw* is the time window. In Figure 6 the same trace detected by the Bio2Bit Move related to the contraction of a specific muscle (lasting three seconds) and repeated with progressively reduced amplitude, is processed, as described above. The timing of the muscle activity is shown with the red line: in Figure 6a, the time intervals during which the contraction is in ON state are indicated by “1”, while the OFF state intervals are indicated by “0”. The muscle activity is correctly detected with progressive amplitude reduction, down to a factor of ten. In that figure, the time window for the threshold definition was *tw =* 500 ms. In Figure 6b, the filtered EMG raw signal is sketched in the ON and OFF time intervals (red dashed line). Finally, in Figure 6a, the blue line represents the signal RMS, while the green line represents the dynamic factor *j*: the former is calculated with a *tw =* 10 ms, the latter with a *tw* = 200 ms.

## 3. Results

**Figure 7:**
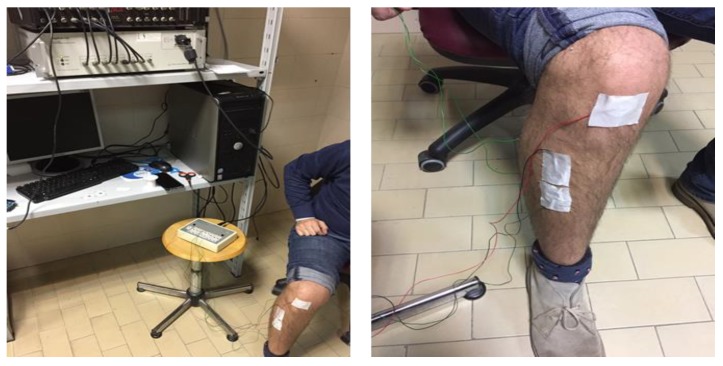
Typical set-up of a sEMG with a conventional wired equipment that is used in biomedical research laboratories.

In order to calibrate our wearable device, we used state-of-art conventional wired equipment (Digitimer D360, Digitimer Ltd., Hertfordshire, UK). In Figure 7, pictures of the Digitimer D360 set-up are shown. As one can see, a number of wires limits and obstacles movements, therefore the calibration tests were performed on sitting subjects. The success of the calibration tests is necessary to allow for us to use the wearable device in subjects moving with wide changes of body/limb position.

Following the indications by SENIAM, the two positive and negative electrodes of the wired equipment were placed along the muscle to be studied, the third reference one far from it.

**Figure 8:**
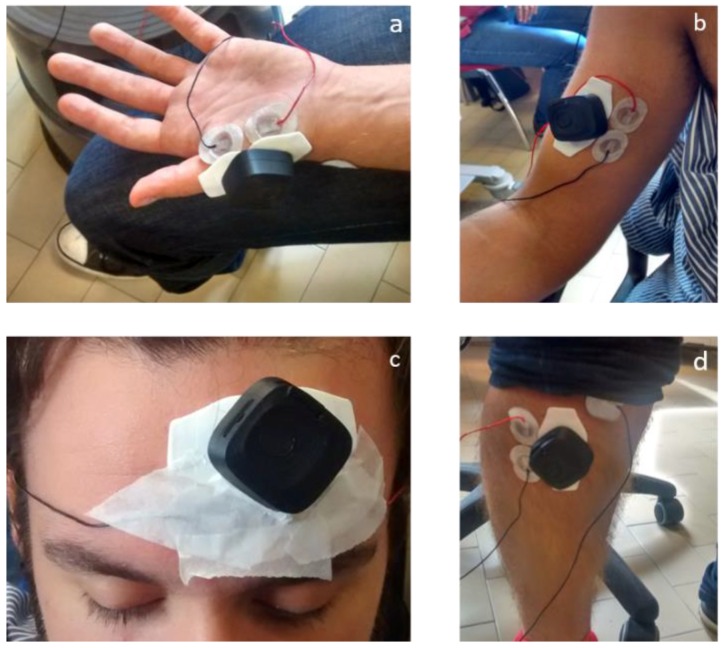
Placement of Digitimer D360 and Bio2Bit surface electrodes on the: (**a**) abductor pollicis brevis muscle; (**b**) biceps brachii muscle; (**c**) frontalis muscle; and (**d**) tibialis anterior.

We performed systematic calibration recording the sEMG signals with the Digitimer D360 and with our wearable device, at the same time, on the same muscle. In general, artefacts due to the presence of skin hair and humidity, adipose tissues, as well as bad patch adhesion, are critical issues for sEMG signal acquisition. To overcome the problem, in our tests, we adopted specific strategies for electrode fixing, consisting of local cleaning and epilation of the skin surface, and ensured the robustness of the patch adhesion. In each test, the electromyograms that were obtained with the Digitimer D360 equipment were our control traces. As shown in Figure 8, the two Bio2Bit Move electrodes were positioned as close as possible to the two signal electrodes of the wired equipment. The contraction activity of abductor pollicis brevis, biceps brachii muscle, tibialis anterior, frontalis muscle, and gastrocnemius (picture not shown for brevity) was monitored.

The traces of the Digitimer D360 and the Bio2Bit Move were displayed on a PC, using the integrated Bluetooth facility for the wearable device.

**Figure 9:**
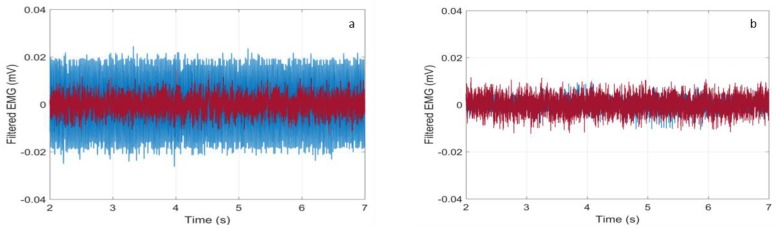
Zoom of the baselines of the wearable device (red line) and the conventional wired equipment (blue line) (**a**) without Notch filter and (**b**) with the Notch filter. The traces refer to a rest time interval in a test on the abductor pollicis brevis.

**Figure 10:**
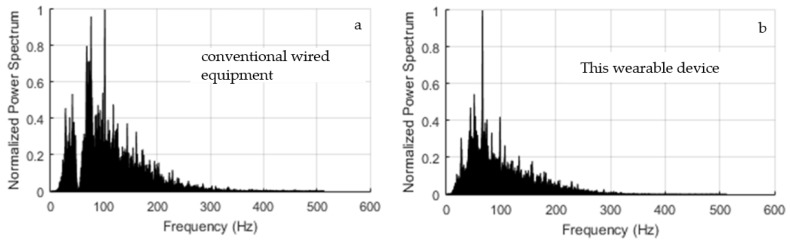
Normalized power spectrum of the sEMG signal during the contraction of the abductor pollicis brevis recorded by: (**a**) Digitimer D360 with Notch filter activated, (**b**) our wearable device.

Preliminary, we noticed that the trace recorded by the Digitimer D360 exhibited a much worse baseline due to the connection to the electrical grid. In fact, in Figure 9a, there are displayed the synchronized baseline traces referring to a rest time interval between 2 and 7 s in the test on the abductor pollicis brevis, as discussed in the next Section. As one can see, the blue trace (Digitimer D360) features a much higher amplitude than the red one (Bio2Bit Move), whereas in Figure 9b, where the Notch filter at 50 Hz of the Digitimer was activated, the two traces are superimposed. Of course, the filter activation penalizes the analysis of contributions around 50 Hz, as it can be verified in Figure 10a. In that figure, we show the normalized power spectrum of the signal that was recorded by the Digitimer during a contraction of the abductor pollicis brevis with the Notch filter activated. It can be compared with the spectrum recorded by our wearable device displayed in Figure 10b. In consideration of the penalty introduced by the Notch filter exactly in the mid of the meaningful band, it was deactivated in all of the tests.

Acquisition of the baseline with the Bio2Bit Move was repeated several times on each subject removing and repositioning the electrodes every time and changing the electrodes every two–three times, resulting in the superposition of all the baselines, thus confirming the repeatability. Obviously, moving from one subject to another, different baselines were found due to specific tissue and skin features.

**Figure 11:**
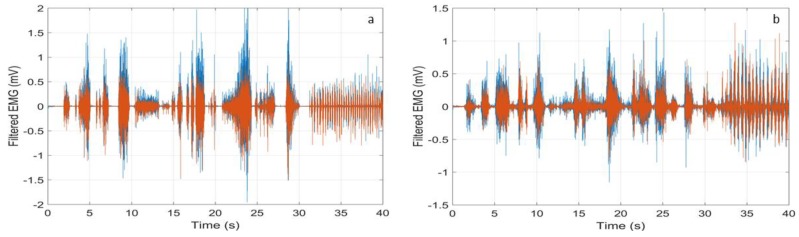
Filtered sEMG signal during random contractions of the tibialis anterior (**a**) and the gastrocnemius muscle (**b**) recorded by the Digitimer D360 with the Notch filter deactivated (blue curve) and by our wearable device (red curve).

Then, we studied the sEMG traces that were recorded during contractions of random duration and intensity (hereafter indicated as “random contractions” for brevity). Four healthy subjects were studied. All of the subjects gave a written informed consent, and the experimental procedures have been approved by the institutional review board of Sapienza University of Rome, Italy, in agreement with the Declaration of Helsinki, and the protocol was approved by the Ethics Committee with Project number 4310. In Figure 11, typical HP filtered signals recorded on the tibialis anterior (a) and the gastrocnemius muscle (b) are shown. The red trace refers to our wearable device and the blue one is the reference trace that was recorded by the Digitimer D360. As one can see, the two traces are superimposed on the time scale. Furthermore, the wearable device records all of the muscle contractions without introducing any erroneous contribution.

**Figure 12:**
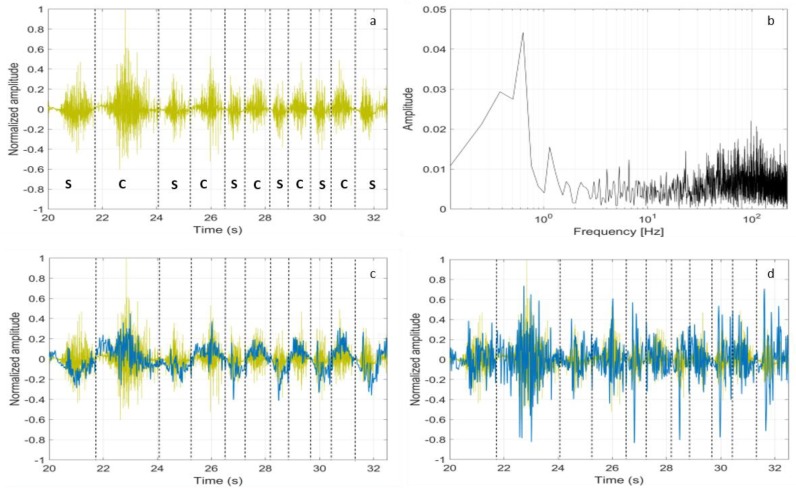
(**a**) Raw sEMG signal during a random stretching and contraction activity of the tibialis anterior recorded by our wearable device. The dashed trace defines the exact timing of stretching (s) and contraction (c); (**b**) FFT of the raw signal; (**c**) The blue trace is the low pass filtered sEMG signal (cutoff at 10 Hz); and, (**d**) The blue trace is the band pass filtered sEMG signal (3–10 Hz).

Starting from the raw sEMG signals, we tried to distinguish the activity of stretching from the contractions. The subjects were asked to alternate the stretching and contractions of the tibialis anterior, while sitting with the foot raised and moving the tiptoe back and forth. In Figure 12a, the raw sEMG signal that was recorded by the Bio2Bit Move is displayed (the dashed lines indicate the stretching (s) and contraction (c) time intervals). As expected, we can see that the raw sEMG signal does not allow for distinguishing contractions from stretching. In Figure 12b, the signal Fast Fourier Transform (FFT) is shown. Starting from that spectrum and following [39], we low pass (LP) filtered the raw signal with a cutoff frequency of 10 Hz without HP filtering, thus including muscle artifacts. The result is shown in Figure 12c, where the LP filtered signal trace (blue) is superimposed to the raw signal (yellow): now, the stretching and concentric contraction activities correspond, respectively, to the negative and positive values of the blue curve. Activating the HP filter degrades the possibility to distinguish the two opposite activities, as it can be seen in Figure 12d, where the blue curve now is the sEMG signal band pass that is filtered in the range 3–10 Hz. In this test, the muscle artifact itself probably helps in distinguishing stretching from contractions.

## 4. Discussion

After having demonstrated that the wearable device correctly detects all of the muscle activity, we applied specific test protocols depending on the studied muscle, which cover a wide range of experimental conditions. As an example, in Table 1, the two protocols relative to biceps brachii and abductor pollicis brevis are described. Referring to it: the muscle was in “Rest” when completely relaxed; “MVC” stands for Maximum Voluntary Contraction; the arm was in “Anti-gravity posture” when folded bearing or not a weight; and, “Isometric contraction against a load” refers to the thumb opposing to a prolonged resistance (load).

**Figure 13:**
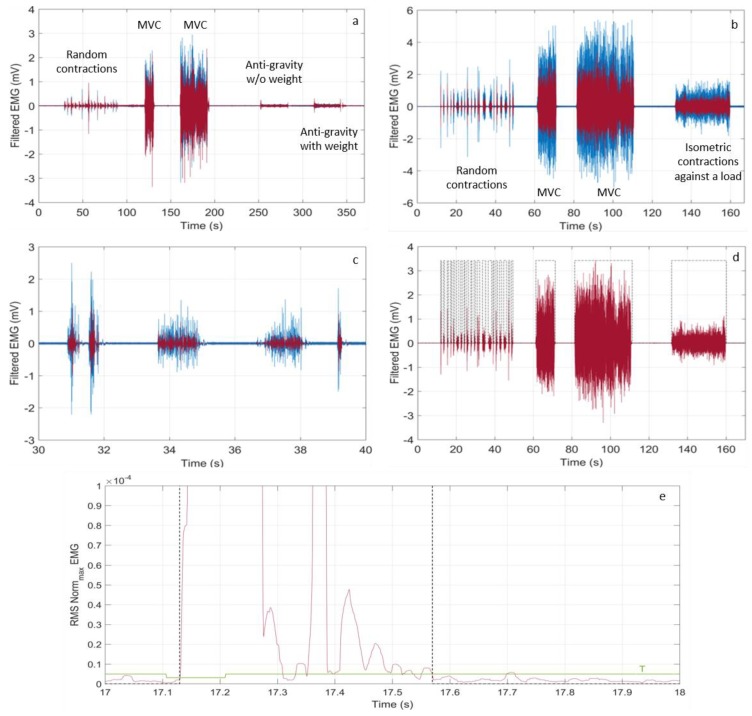
Filtered sEMG traces recorded with the wearable device (red line) and the Digitimer D360 (blue line) in the case of (**a**) biceps brachii contractions and (**b**) the abductor pollicis brevis; (**c**) zoom of the repeated fast and sudden random contraction of the biceps brachii in the time interval between 30 s and 40 s; (**d**) classification of ON state interval times (shadowed black line) of the abductor pollicis brevis activity; and, (**e**) zoom of the ON classification (black dashed line) of the abductor pollicis brevis and the threshold T (green line).

Results are shown in Figure 13a,b.

To get reliable clinical and physiological information, our system should detect timely the onset and the end of any muscle activity. In Figure 13c, a zoom of the sEMG signal relative to the biceps brachii between 30 s and 40 s is displayed. Classification of the ON state of the abductor pollicis brevis activity is shown in Figure 13d, while in Figure 13e, a single ON classification on the signal RMS (between 17 s and 18 s) is reported with the dashed black line, together with the dynamic threshold T, which defines the ON state. All of these results put in evidence that the detection and classification capabilities of our system are robust with respect to the eventuality of very short and weak muscle activity, featuring a time resolution around 60 ms and a minimal detected amplitude of around 100 μV. Similar results were obtained also in tests that were performed on other muscles.

We quantified the performance of our system, calculating the accuracy, the sensitivity, and the specificity on a statistical sample that was made of 94 recordings on four healthy subjects, including weak, intense, very short, and very long muscle activities. The overall recording time was 1750 s. The measurements were performed removing, repositioning and changing the electrodes. We here defined as *true positive* the contraction (ON) time and as *true negative* the inactivity (OFF) time, both being detected by the conventional wired equipment. The sensitivity was evaluated by calculating the true positive respect to the sum of the ON time detected and the ON time *not* detected by the Bio2BitMove. Then, we evaluated the specificity, calculating the true negative respect to the sum of the OFF time and the false ON time that was detected by the Bio2BitMove. We obtained an accuracy of 100% in detecting contraction events with an amplitude higher than 100 μV. The same 100% accuracy in the detection of the contraction events was also reported by another wearable system proposed very recently in literature [40]. Far beyond that, our system features more meaningful achievements as a sensitivity and a specificity both over 80%, calculated on the exact time duration of the contractions, not simply on the detection of the event, but also in the case of muscle activity time intervals as short as some tens of ms. The values of percentage sensitivity and specificity listed in Table 2 are the average over the 1750 s total recording time. The starting latency and the ending latency were also calculated. The starting latency is defined as the difference between the starting edges of ON state evaluated while considering the raw signal of the Digitimer and the raw signal of the Bio2Bit Move; the ending latency is defined as the difference between the ending edges of ON state evaluated when considering the raw signal of the Digitimer and the raw signal of the Bio2Bit Move. Values of the percentage starting latency and the ending latency averaged over the total ON state events are listed in Table 2.

Finally, we wish to make some considerations about the power consumption of our portable device. The algorithm takes 41 ms for processing 104 samples that were acquired at 512 Hz, corresponding to a 208 ms signal time interval. This is approximately 20% of the CPU load of the ARM^®^ Cortex^®^-M4 at 32 MHz, using 25 KBytes ROM and 16 KBytes RAM. In these conditions, the wearable device dissipates only 26 mW in monitoring operation (i.e., processing data in real-time and saving them on the memory). Since the device battery features 592 mWh, this means that, without Bluetooth transmission, the system has more than 20 h operation autonomy, largely covering one day activity. This outlines that our wearable device features outstanding performance and that it can serve for reliable autonomous use by persons during day-life, both outdoor and indoor.

## 5. Conclusions

In conclusion, the proposed wearable device is a stand-alone system-in-package that performs real-time monitoring of the muscle activity sensing and processing autonomously the electromyography signal without any external device. The algorithms for data processing are embedded in the integrated low-power microcontroller. Systematic tests of muscle contraction detection demonstrated that our system performs comparably to state-of-art wired equipment that is conventionally used in biomedical research laboratories with the advantage of distinguishing between muscle stretching and contraction. Low and short muscle activity with an amplitude level down to 100 µV and time interval as short as approximately 60 ms are correctly recognized. We studied a statistical sample of approximately one hundred recordings on four subjects in a wide range of experimental conditions, including maximum voluntary contractions, anti-gravity postures with and without a weight, and isometric contractions against a load. As a final result, our system achieved specificity and sensitivity in recognizing exact activity timing over 87% and 82%, respectively, with the advantage of being wireless and comfortably wearable.

The system exhibits a power consumption as low as 26 mW in monitoring operation, reflecting in a long autonomy of a conventional 592 mWh battery, well adapting to a daily use, outdoor, or indoor.

Finally, the possibility of integrating several functionalities directly in the device opens to the future convergence of multimode operations on the same platform, with the contemporary detection of different physiological and physical signals. For all those reasons, this wearable device candidates to represent a helpful tool for domestic and outdoor use, capturing data with far greater ubiquity, regularity, and comprehensiveness than has been traditionally possible.

## Figures and Tables

**Table 1:** Description of the procedures of two typical tests.

Brachial Biceps	Abductor Pollicis Brevis
Rest (30 s)	Rest (10 s)
Random short contractions (60 s)	Random short contractions (40 s)
Rest (30 s)	Rest (10 s)
MVC (10 s)	MVC (10 s)
Rest (30 s)	Rest (10 s)
Long MVC (30 s)	Long MVC (30 s)
Rest (60 s)	Rest (20 s)
Anti-gravity posture (30 s)	Isometric contractions against a load (30 s)
Rest (30 s)	Rest (10 s)
Anti-gravity posture with ½ Kg weight (30 s)	
Rest (30 s)	

**Table 2:** Percentage values of sensitivity and specificity averaged over the total recording time; starting latency and ending latency averaged over all of the ON state events.

Sensitivity %	Specificity %	Starting Latency %	Ending Latency %
87.18	82.60	0.33	2.33
